# Bioprosthetic valve monitoring in patients with carcinoid heart disease

**DOI:** 10.3389/fcvm.2022.1072890

**Published:** 2023-01-12

**Authors:** Kevin A. Honan, Saamir Hassan, Anita Deswal, Joerg Herrmann, Juhee Song, Dominique Monlezun, Daniel Halperin, Armeen Mahvash, Arvind Dasari, Efstratios Koutroumpakis, Mehmet Akay, Dinu-Valentin Balanescu, Ismael Salas de Armas, Manish Patel, Sriram Nathan, Biswajit Kar, Konstantinos Marmagkiolis, Juan Lopez-Mattei, Jay Patel, Igor Gregoric, James Yao, Cezar A. Iliescu

**Affiliations:** ^1^Department of Internal Medicine, McGovern Medical School, The University of Texas Health Science Center at Houston, Houston, TX, United States; ^2^Department of Cardiology, The University of Texas MD Anderson Cancer Center, Houston, TX, United States; ^3^Department of Cardiovascular Medicine, Mayo Clinic, Rochester, MN, United States; ^4^Department of Gastrointestinal Medical Oncology, The University of Texas MD Anderson Cancer Center, Houston, TX, United States; ^5^Division of Diagnostic Imaging, Department of Interventional Radiology, The University of Texas MD Anderson Cancer Center, Houston, TX, United States; ^6^Department of Cardiothoracic Surgery, McGovern Medical School, The University of Texas Health Science Center at Houston, Houston, TX, United States; ^7^Center for Advanced Heart Failure, Memorial Hermann Hospital, Heart and Vascular Institute, Texas Medical Center, Houston, TX, United States

**Keywords:** cardio-oncology, carcinoid heart disease, telotristat ethyl, peptide receptor radionuclide therapy (PRRT), pulmonary valve, tricuspid valve repair, tricuspid valve (TV), pulmonary valve (stenosis) (insufficiency)

## Abstract

**Background:**

Carcinoid heart disease (CnHD) is a frequent cause of morbidity and mortality in patients with neuroendocrine tumors and carcinoid syndrome. Although valve replacement surgery appears to decrease all-cause mortality in patients with advanced CnHD, few studies have investigated the outcomes of patients after valve replacement.

**Methods:**

We conducted a multi-institution retrospective registry of patients who received both tricuspid and pulmonic bioprosthetic valve (TV/PV) replacements for advanced CnHD from November 2005 to March 2021. Patients were followed post-operatively with echocardiographic studies every 3 months. Carcinoid valvular heart disease scores were used to monitor valve degeneration. Neuroendocrine tumor treatment, their administration times, and associations with echocardiographic findings were recorded.

**Results:**

Of 87 patients with CnHD, 22 patients underwent simultaneous surgical TV and PV replacement. In 6 patients (27.3%), increased PV V_max_ was the first echocardiographic manifestation of valve degeneration in the setting of occult neurohormonal release. Post-operative telotristat ethyl and peptide receptor radionuclide therapy appeared to stabilize PV V_max_. The PV V_max_ showed consistent elevation in the entire patient population when compared to baseline, while bioprosthetic TV echocardiographic parameters were relatively unchanged throughout. Post-operative warfarin therapy did not affect the rate of PV degeneration, and no major bleeding was recorded during or after post-operative anticoagulation therapy.

**Conclusion:**

Bioprosthetic valve degeneration is common in CnHD. Monitoring with echocardiographic studies every 3 months, focusing on PV velocities, could identify patients with occult disease that very likely promotes valve degeneration. Novel neuroendocrine tumor therapies may have a beneficial impact on valve degeneration.

## Introduction

The incidence of neuroendocrine tumors (NETs) is on the rise (1) with a 2017 analysis estimating that, in the United States, over 170,000 individuals have NETs (2). Although the reported rates of carcinoid syndrome (CS) in the NET population have varied from 3 to 6% in smaller case series, a large, population-based analysis using the Surveillance, Epidemiology, and End Results-Medicare database reported a CS rate of 19% (3, 4). In this analysis, rates varied by primary site and stage, with rates being highest among patients with distant tumors of the terminal ileum/cecum (52%) and small intestine (56%) (3). Reports suggest that over half of patients with these distant tumors will develop carcinoid heart disease (CnHD) (5–9). The current guidelines for treating advanced CnHD primarily recommend valve surgery (10), the use of somatostatin analogs, and cardiac symptom palliation (e.g., treating peripheral volume overload in right-sided heart failure with standard loop and/or thiazide and aldosterone receptor blocker diuretic therapy; neurohormonal blockade and digoxin with limited proven value in these patients) (9, 11).

Although valve replacement surgery in patients with advanced CnHD has shown an all-cause mortality reduction (10), no post-operative monitoring studies have evaluated outcomes in CnHD patients managed with surgical bioprosthetic valve replacement (8, 12). While morbidity and mortality of bioprosthetic valve replacement continue to improve in the general population, bioprosthetic valve degeneration after surgery remains an ongoing concern for patients with CnHD (10). This is the first study to systematically monitor tricuspid and pulmonic valves after bioprosthetic valve replacement and report the impact of early intervention using novel NET systemic therapies.

## Materials and methods

### Study population

**FIGURE 1:**
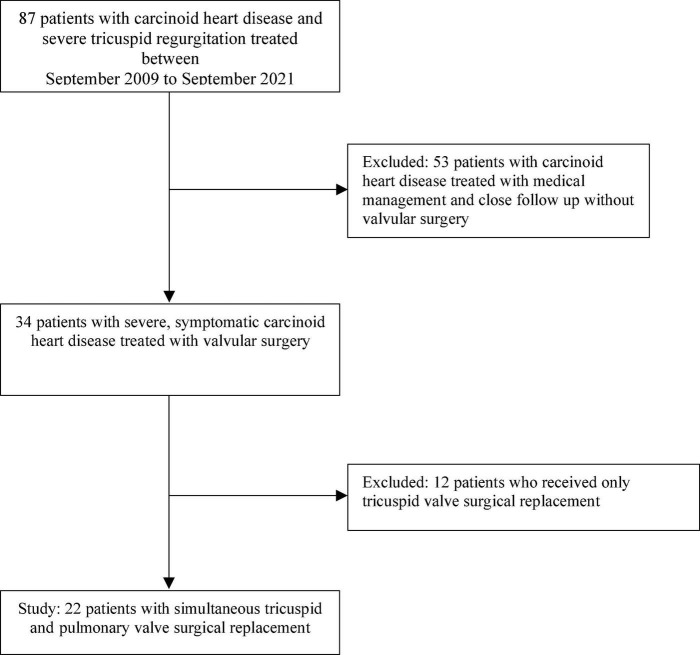
Inclusion and exclusion criteria for the study.

This registry included all adult patients with biopsy-proven NET with carcinoid syndrome and severe CnHD who received valve replacement from November 2005, through March 2021 (Figure 1). Patients were enrolled in a scheduled monitoring program with echocardiographic evaluation every 3 months. All patients underwent at least simultaneous surgical TV and PV replacement for CnHD. All tricuspid valve bioprosthetic valves were St. Jude EPIC valves, ranging from 27 to 31 mm. Pulmonary bioprosthetic valves were mostly St. Jude EPIC (range 21–25 mm); Two were Edwards Inspiris, and one was Edwards Sapien. Five patients had St. Jude EPIC SUPRA bioprosthetic aortic valve replacement and one received a St. Jude EPIC mitral valve prosthesis. Patients with milder CnHD (i.e., who required only TV replacement) and those who received medical management alone for their CnHD were excluded from the study. MD Anderson Cancer Center’s Institutional Review Board approved the protocol, and informed consent was waived because of the retrospective nature of the study.

Patients underwent transthoracic echocardiography prior to and 3 months after their valve surgery; all patients underwent scheduled follow-up transthoracic echocardiography every 3 months thereafter. Standard echocardiographic equipment (Philips Medical Systems, NA, Bothell, Washington; General Electric Medical Systems, Milwaukee, Wisconsin; and Siemens Medical Solutions USA, Inc., Malvern, Pennsylvania) was used for this purpose. In the pre-surgical and follow-up echocardiograms, the left ventricular ejection fraction was calculated using the Simpson biplane method of disks per European Association of Cardiovascular Imaging and American Society of Echocardiography recommendations (13). Right-sided valve dysfunction was graded according to American Society of Echocardiography guidelines and carcinoid valvular heart disease (CVHD) scoring parameters (13, 14). CVHD scoring parameters were performed in patients after valve replacement surgery, though this scoring system has not been previously utilized in the post-operative setting (14).

Patients were defined as having “occult disease” when subclinical valve changes were observed in the setting of controlled typical carcinoid vasomotor symptoms (flushing, diarrhea, and/or anxiety) and stable tumor size.

### Clinical data collection

Baseline demographics were obtained for all patients at the time of cardiac catheterization that preceded the valve surgery. Patients were enrolled in a monitoring program that included clinical evaluation and scheduled echocardiographic studies every 3 months post-operative. Outcome data were collected with manual chart review of electronic medical records.

### Echocardiographic imaging and data

The American College of Cardiology/American Heart Association expert statement on CnHD indicates that transthoracic echocardiography (TTE) should be performed in all patients with carcinoid syndrome who have high suspicion of CnHD, those with any change in clinical status, or routinely every 3–6 months, depending on CnHD severity (9).

The last echocardiographic study prior to valve surgery and the velocities recorded at that time represented the “pre-operative echocardiogram” for this study. The post-operative echocardiographic study at 3 months was considered the “baseline study” followed by scheduled echocardiographic studies and clinic visit every 3 months. All administered cardiac or oncological medications were recorded and summarized.

Valve peak velocity (V_max_) and velocity- time integrals (VTI) were calculated using continuous-wave (CW) Doppler and pulse-wave (PW) Doppler echocardiography, respectively. At the time of each echocardiographic study, right and left ventricular systolic function, and blood pressure and heart rate were assessed.

All patients had severe TV regurgitation and pulmonic valve involvement (at least moderate regurgitation and/or stenosis). Symptomatic CnHD was considered when patients had fatigue and/or dyspnea, lower extremity peripheral edema, pleural effusion, ascites, or any combination of these symptoms. The degree of TV regurgitation and PV regurgitation/stenosis were defined according to current echocardiographic guidelines and confirmed by two independent cardiologists (15).

Lastly, the carcinoid valvular heart disease (CVHD) scoring method by Denney et al. was adapted to each patient’s echocardiography profile (14); this method–though a pre-surgical surrogate of CnHD–was chosen to best illustrate the status of the native TV and PV before, and status of the bioprosthetic replacements after surgery. There score’s four main components included: TV anatomy, TV regurgitation severity, PV stenosis severity, and PV regurgitation severity. Each of the four categories were given a corresponding numerical score based on the weighted guideline set forth by Denney et al. (14), and a *CVHD%* score was given to each patient for each of their echocardiogram study. Expanding further upon the PV components of the CVHD score, we chose to mark patients with an increase of 20% PV V_*max*_ in two consecutive studies as having a noteworthy increase in hemodynamics suggesting (bioprosthetic) degeneration.

### Surgical procedure

During surgery patients were placed on cardiopulmonary bypass and the beating-heart technique for valve replacement was utilized. The TV was inspected first. The anterior leaflet was excised, and the valve was measured before an Epic prosthesis was fitted. Next, the pulmonary artery was opened longitudinally, and the PV was inspected. The surgeon measured the valve and then excised the cusps before implanting the PV prosthesis into the orifice of the PV annulus. Additional left sided affected valves (aortic and mitral) were replaced as indicated and included in the post-operative monitoring program. All patients underwent pre-operative and intraoperative transesophageal echocardiography (TEE), with excellent correlation with pre-surgical transthoracic data. The same limitation as in transthoracic echo was exhibited in pulmonic valve visualization due to decreased forward flow.

### Outcomes measurement

Survival was measured in days after valvular surgery. Any PV V_max_ increase greater than 20% observed in two consecutive echocardiographic studies, when compared to the 3-month follow-up echocardiogram (to allow biventricular remodeling), triggered additional advanced treatment of the underlying carcinoid disease while quarterly echocardiographic monitoring continued. Despite the initiation of anticoagulation upon observation of increased velocities, PV velocity continued to increase, triggering concern about uncontrolled neurohormonal disease rather than a valvular thrombotic event. Due to limited visualization of the pulmonic valve on the echocardiographic studies, clinical decision were based solely on the PV velocities. Survival status and the most recent follow-up date were determined from patient electronic medical records.

### Statistical analysis

Patients’ baseline demographics and pre- and post-surgical echocardiography parameters were summarized by descriptive statistics, with means (±the standard deviation) or medians (with interquartile ranges) used for continuous variables and frequencies (with percentages) used for categorical variables. The pre-operative echocardiographic parameters were compared with the post-operative and 3 months (baseline) follow-up echocardiographic parameters using the paired *t*-test or Wilcoxon signed rank test, as appropriate. *P-*values of less than 0.05 were considered statistically significant. For the comparisons of echocardiographic parameters, Bonferroni adjusted alpha (0.05 divided by 11 = 0.0045) was considered as well. The overall survival time was defined as days until death or last known follow up after the initial heart surgery, and the survival probability was estimated using the Kaplan–Meier method. A Cox regression model with a time-varying covariate was used to evaluate the effect of post-surgical treatment on overall survival. Extended Kaplan–Meier method was used to illustrate the association between post-surgical treatment (as a time varying covariate) and mortality (16). Extended Kaplan–Meier curves represent hypothetical cohorts whose post-surgical treatment statuses remain constant throughout follow-up (so they are not to be interpreted as the estimated probabilities of an event for each cohort over time) (17, 18). Profile plots of echocardiographic parameter values were plotted over time for each patient.

## Results

From November 2005, through March 2021, 87 patients had CnHD and 34 patients were deemed surgical candidates; 22 of these 34 patients got simultaneous tricuspid and pulmonic valve surgical replacement for CnHD. There were 4 patients requiring additional AVR and one underwent replacement of all 4 valves. Patients were predominantly male with the majority being without cardiovascular comorbidities (i.e., without diabetes mellitus, hyperlipidemia, coronary artery disease, or prior congestive heart failure; see Table 1). All patients were treated with somatostatin analogs (octreotide or lanreotide) before surgery at doses adjusted by the primary oncology team to achieve symptom control and radiographic tumor stabilization; patients were admitted to the hospital at least 24 h prior to their surgery and initiated on IV octreotide bolus and maintenance infusion (50–100 mcg IV; 50 mcg/hour). Patients were given an additional bolus during surgery and continued IV maintenance therapy after surgery for at least 48–72 h. Only one patient had carcinoid crisis during induction: twice, despite incremental doses of octreotide drip and boluses, requiring additional liver embolization and addition of telotristat ethyl. The third attempt was successful induction and she was asymptomatic during the surgical and post-operative period. Approximately half of the patients received anticoagulants after surgery (Table 2).

**TABLE 1:** Patients’ baseline characteristics.

Covariate	Value (*N* = 22)
**Sex, *n* (%)**	
Male	14 (63.6)
Female	8 (36.4)
**Race, *n* (%)**	
Non-Hispanic White	12 (54.5)
Black	6 (27.3)
Hispanic	4 (18.2)
Body mass index, mean ± SD	25.44 ± 4.21
Tobacco use in the last year, *n* (%)	1 (4.5)
Hypertension, *n* (%)	16 (72.7)
Dyslipidemia, *n* (%)	3 (13.6)
Diabetes, *n* (%)	2 (9.1)
Coronary artery disease, *n* (%)	1 (4.5)
Peripheral arterial disease, *n* (%)	1 (4.5)
Family history of carcinoid disease, *n* (%)	4 (18.2)
Congestive heart failure, *n* (%)	6 (27.3)
Atrial fibrillation, *n* (%)	3 (13.6)
Stroke, *n* (%)	1 (4.5)
Chronic lung disease, *n* (%)	0 (0)
**Laboratory values at the time of surgery, mean ± SD**	
Aspartate aminotransferase (units/L)	38.5 ± 17.23
Alanine transaminase (units/L)	39.09 ± 38.31
Albumin (g/dL)	3.58 ± 0.58
International normalized ratio	1.25 ± 0.22
Urinary 5-hydrooxyindoleacetic acid (mg/day)	199.09 ± 111.66
Chromogranin A* (ng/L)	1726 (837–7563)
Neuron-specific enolase (ng/mL)	17.55 ± 14.83
N-terminal pro-B-type natriuretic peptide (pg/mL)	309.86 ± 176.13
White blood cells (K/uL)	5.65 ± 3
Platelets (K/mcL)	225.36 ± 116.46
Estimated glomerular filtration rate (mL/min/1.73 m^2^)	74.13 ± 30.49

*Median (interquartile range). Values of continuous variables are shown as mean ± SD; SD, standard deviation.

**TABLE 2:** Patients’ treatment regimens.

Type of carcinoid symptom control	Medication	*n* (%) *N* = 22
Pre- and post-surgical symptom control	Octreotide	16 (72.7%)
	Lanreotide	6 (27.3%)
Post-surgical inhibition of tryptophan hydroxylase	Telotristat ethyl	6 (27.3%)
Molecular targeted therapy	PRRT with Lu-177	5 (22.7%)
Anticoagulation	Warfarin	12 (54.5%)

Lu-177, lutetium oxodotreotide; PRRT, peptide receptor radionuclide therapy.

**FIGURE 2:**
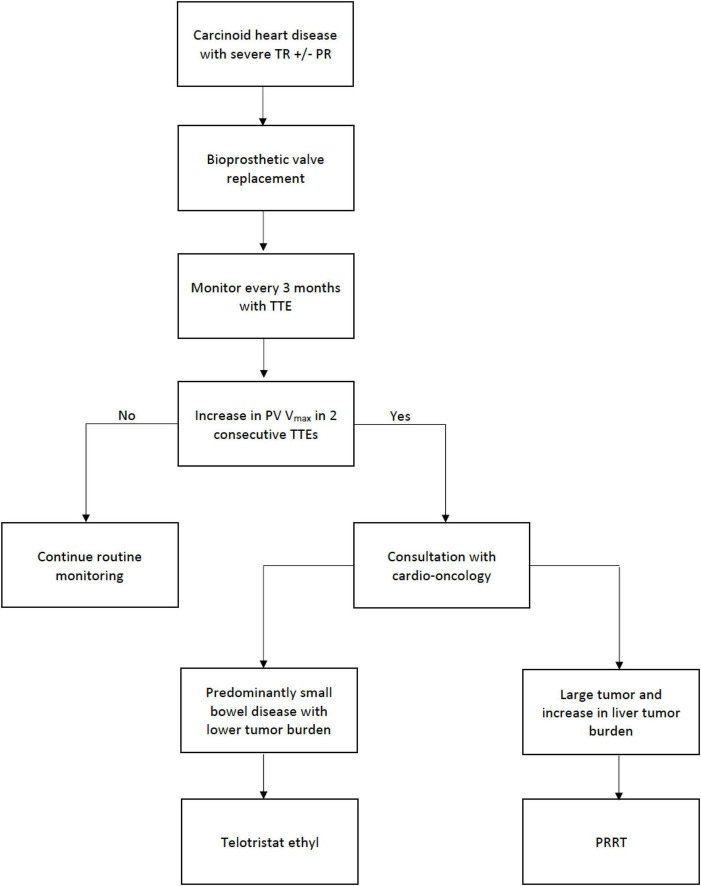
Post-surgical treatment protocol.

Increased velocities across the PV noted in two consecutive post-surgical monitoring echocardiographic studies were recorded in 6 patients (27%). For these 6 patients, telotristat ethyl treatment was the first line of treatment in patients deemed to have low tumor burdens, while peptide receptor radionuclide therapy (PRRT) was used in patients that had large tumor burden (19, 20) (Figure 2). PRRT was also added to patient treatment regimens as a supplement when valvular deterioration was not controlled with telotristat ethyl alone. Our first patient who received advanced treatment with telotristat was in July 2017 (note that telotristat was approved by the FDA in 2017), while the first patient from our study treated in PRRT was 2 years before FDA approval, in 2016, on a compassionate basis (PRRT treatment was approved by the FDA in 2018). Patients needing advanced therapies had discussions with the cardio-oncology team regarding eligibility and the results from the NETTER-1 trial showing significant increase in progression-free survival in patients with carcinoid tumors.

Compared to the pre-operative echocardiograms, the immediate post-operative follow-up echocardiograms showed an expected improvement in the CVHD score, TV peak velocity, and TV peak gradient (all *p*-values < 0.05). The first official post-operative follow up echo (done at 3 months post-op) marked the patient’s new “baseline echocardiogram,” accounting for a period of right ventricular function recovery and remodeling. At this point, the mean CVHD score, TV peak velocity, TV peak gradient were lower than the baseline values indicating improved heart function; changes in systolic blood pressure were not statistically significant. Mean PV V_max_, PV peak gradient, and PV mean gradient values were higher than the immediate post-operative values (all *p*-values < 0.05; see Table 3). At the second follow-up (6 months post-op), the mean CVHD score and diastolic blood pressure values were again lower than the baseline values; however, the mean PV peak velocity, PV peak gradient, and PV mean gradient values continued to rise.

**TABLE 3:** Comparison of cardiac measurements before and after surgery.

Covariate		Pre-operative	Post-operative	Difference	
	*n*	Mean ± SD	Mean ± SD	Mean ± SD	*P-*value^1^
LV EF^2^	21	60 (55–65)	63 (60–65)	3.08 (−1–5)	0.2680^3^
CVHD	17	78.15 ± 10.85	37.82 ± 5.51	−40.34 ± 13.35	**<0**.**0001**
TVpeak velocity	21	2.28 ± 0.5	1.58 ± 0.38	−0.7 ± 0.51	**<0**.**0001**
TVpeak gradient	21	22.26 ± 8.68	11.3 ± 6.01	−10.96 ± 8.92	**<0**.**0001**
TVmean gradient^2^	21	7.2 (4.3–10.8)	4.2 (3.8–6)	−0.6 (−7.6–1.5)	0.0817^3^
PV peak velocity	21	1.81 ± 0.48	2.42 ± 0.86	0.61 ± 0.9	**0**.**0059**
PV peak gradient	21	14.06 ± 6.92	26.04 ± 14.99	11.98 ± 15.97	**0**.**0026**
PV mean gradient	20	6.86 ± 3.27	14.76 ± 9.45	7.9 ± 9.43	**0**.**0014**
Systolic blood pressure^2^	18	124 (109–142)	113.5 (108–117)	−5.5 (−26–2)	0.0519^3^
Diastolic blood pressure^2^	18	74.5 (72–80)	69 (60–77)	−4.5 (−9–3)	0.0578^3^
Heart rate	19	76.95 ± 13.53	80.32 ± 15.84	3.37 ± 14.42	0.3219

^1^Paired *t*-test is used unless specified.

^2^Median (IQR) are presented instead of Mean ± SD.

^3^Wilcoxon signed rank test is used.

Post-operative: Post-operative TTE done immediately in post-op window within the first week of surgery.

Bold values represent the statistically significant; *p*-value < 0.05.

**FIGURE 3:**
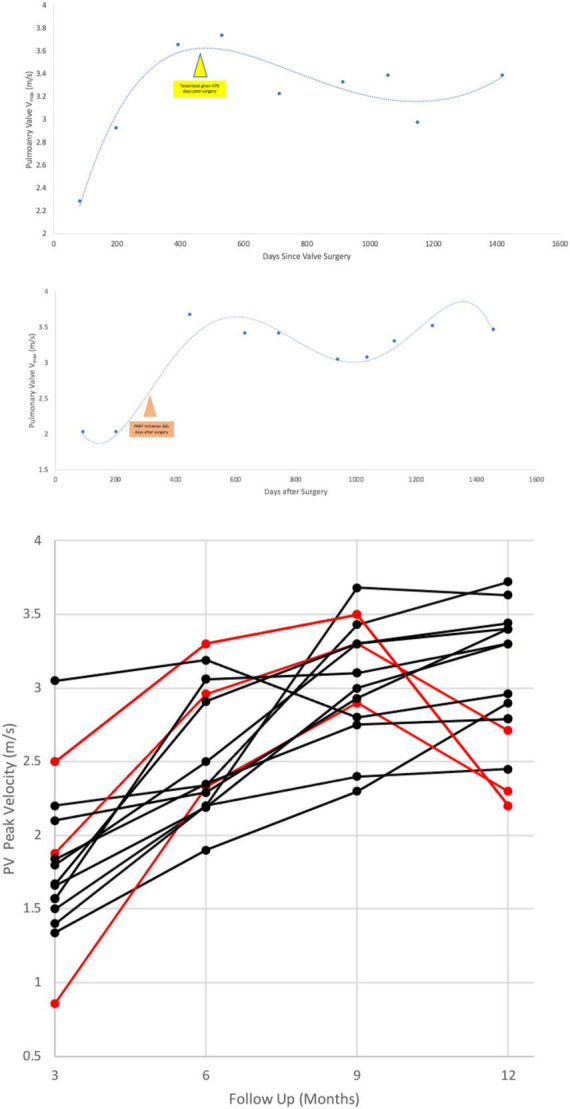
An example of an perioperative rise in pulmonary valve peak velocity after heart surgery in a patient with bioprosthetic TV + PV replacement **(Top, Middle)**. **Bottom** shows the effect of telotristat ethyl treatment with or without peptide receptor radionuclide therapy on post-surgical pulmonary valve velocities. Time 0, x-axis, is at 3 months post-op. Red lines are patients’ PV velocities who were given advanced therapies (telotristat ethyl, PRRT) in this post-operative timeframe. PRRT, peptide receptor radionuclide therapy; V_max_, peak velocity.

The patient’s echo 3 months post-op was defined as the patient’s “baseline” echo. Every study thereafter, was scheduled at 3 month intervals. Six patients had at least a 20% increase in PV V_max_ in two consecutive studies and were treated with telotristat; 2 of these patients additionally received PRRT after treatment with telotristat. Three other patients received PRRT at onset of significant PV V_max_ increase. In both advanced therapies, PV V_max_ plateaued as early as the next scheduled 3 month follow (Figure 3); in some cases there was reversal in the increasing PV V_max_ in the prolonged post-operative period (Figure 3 and Supplementary Figure 1).

**FIGURE 4:**
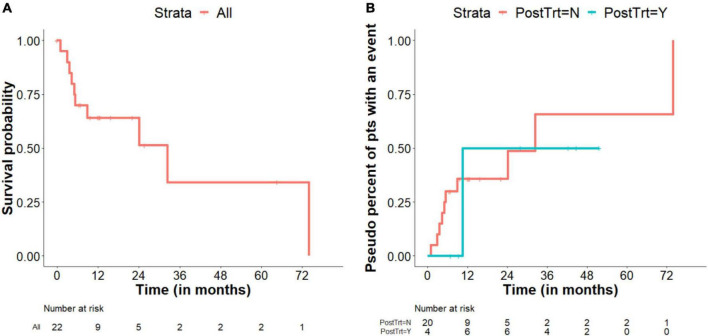
Survival. **(A)** Kaplan–Meier survival plot includes all patients while they were under observation only. Patients who were given post-surgical advanced therapies were censored at the time of treatment initiation. **(B)** Pseudo percent of patients with an event (death) comparing treatment and observation alone, as a time varying covariate. The sizes of the risk sets can increase or decrease over time, depending on start time of telotristat ethyl and/or peptide receptor radionuclide therapy (PRRT); Note that the number at risk at the start of the trial is 4 for those who were treated with telotristat ethyl and/or PRRT. Two patients started telotristat ethyl and/or PRRT right after the first heart surgery. The other two patients started telotristat ethyl and/or PRRT at 6.4 and 6.9 months after the first heart surgery, which is earlier than the first death event in patients treated with telotristat ethyl and/or PRRT.

In contrast, post-surgical warfarin therapy did not seem to affect the rate of PV degeneration. In addition, patients who received post-surgical telotristat ethyl with or without PRRT showed a trend toward increased survival after 12 months than those who did not receive these additional treatments (Figure 4B). Notably, none of the patients who received additional treatments died during the study period.

Bioprosthetic TVs showed no signs of degeneration (tricuspid stenosis and/or tricuspid regurgitation) at all follow-up echocardiographic points with no significant differences in CVHD scoring at 6 months irrespective of treatment with telotristat ethyl or PRRT (Table 3). While TV regurgitation and PV stenosis/regurgitation were the primary pathological characteristics noted prior to surgery, PV stenosis was the primary pathological characteristic seen in degenerated valves after surgery on follow-up echocardiograms.

The median follow-up was 42.4 months (95% CI, 22.1–51.7 months), and the median overall survival was 32.4 months (95% CI, 5.3–74.0 months). A Kaplan–Meier survival plot for all patients under routine post-surgical observation-alone (without advanced post-surgical therapies) is presented in Figure 4A, whereas a pseudo percent of patients with an event (death) for patients according to treatment status (hypothetical cohorts) are shown in Figure 4B. Based on a Cox regression model with post-surgical treatment as a time-varying covariate, HR (95% CI) of post-surgical treatment vs. observation only was 0.370 (0.041–3.332) (*p* = 0.375).

## Discussion

This is the first monitoring study of bioprosthetic tricuspid and pulmonic valves for carcinoid heart disease. This study recognized increases in PV V_max_ as the signal active “occult disease” despite stable tumor burden and controlled carcinoid syndrome symptoms, a phenomenon that might be best explained by recalling the bioactivity of these malignant neuroendocrine tumors: Before their biochemical breakdown, multiple vasoactive substances secreted by NETs, including 5-HT (serotonin), prostaglandins, histamine, bradykinin, tachykinins (substance P, neurokinin A, neuropeptide K), and transforming growth factor-β, drive plaque deposition on the heart valves—predominantly the TV and PV on the right side of the heart—and a pathological cascade associated with worse outcomes in patients with CnHD (21). The cleaving and inactivation of these vasoactive substances in the pulmonary vasculature explains the rarity (<10% of cases) of left-sided heart valve involvement in the general population of CnHD patients without right-to-left interatrial shunting (i.e., patients without patent foramen ovale) or in those with NET lung cancer or metastatic lung disease (22). Advanced heart failure (New York Heart Association Classes III and IV) from right-sided valve degeneration has been associated with high all-cause mortality in large retrospective studies of CnHD (23). Serum levels of serotonin and urinary 5-hydroxyindoleacetic acid levels in NETs directly correlate with clinical symptoms (most commonly flushing, diarrhea, and anxiety) (24, 25).

**FIGURE 5:**
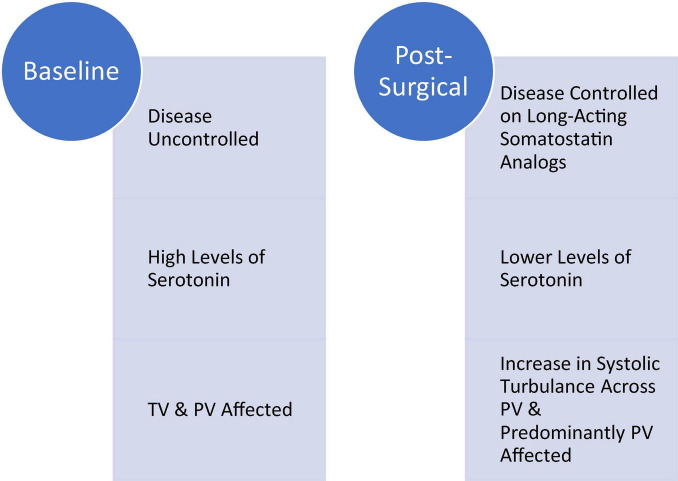
Baseline symptomatic carcinoid heart disease (CnHD) versus post-surgical CnHD.

In the early experience with CnHD, patients reached the point of valve replacement surgery late with poor functional status, low serum albumin levels, and portended suboptimal outcomes (26). This ultimately shifted the approach toward a rather earlier valve replacement. With the advancement in surgical techniques and perioperative care paralleled by the improved pre-surgical patient optimization (mean albumin level in was 3.6 g/dL), we had no perioperative mortality or continuous clinical deterioration post-surgically. In our study, all patients had symptoms controlled with octreotide and lanreotide, none experienced typical carcinoid syndrome symptoms or had recorded changes in their tumor burdens on follow-up computed tomography or magnetic resonance imaging. Despite that, on the monitoring echocardiography we had evidence of bioprosthetic valve degeneration and increase in PV valve velocities (mean difference at first follow up, and beyond: starting with + 0.63 ± 0.74 m/second; see Table 3). This suggested to us that it appears current metrics for biological activity of NETs and disease burden (CT thorax, abdomen and pelvis) may be insufficient, as patient are still having increased mortality and evidence of valve deterioration despite control of their carcinoid syndrome symptoms (Figure 5).

All patients started on the tryptophan hydroxylase inhibitor telotristat ethyl and/or PRRT reported improvement in their functional status and level of activity and were alive at the end of our study and the valvular degeneration appeared to have stabilized. Based on this data we hypothesized the presence of what we have defined as “occult disease” less evident on traditional tumor imaging and not completely captured by the typical carcinoid symptoms that can be identified early with echocardiographic imaging focused on the bioprosthetic valves with additional emphasis on the pulmonic valve. Although the patient numbers when studying rare diseases is small and much remains unknown about these exciting novel therapies, it is plausible that carcinoid symptom control and tumor size, alone, may lack the sensitivity needed to correctly monitor these patients, and that close echocardiographic monitoring added to novel NET therapies can improve the overall patient outcomes. Patients that did not receive treatment with telotristat ethyl and peptide receptor radionuclide therapy had high mortality within the first 12 months after surgery (Figure 4B). Due to sample size, our study did not show a significant mortality difference in observation-only patients versus those receiving telotristat ethyl and/or PRRT, though we expect that a larger sample study could validate the role of the novel NET therapies in preventing bioprosthetic valve degeneration. Current randomized clinical trial in CnHD patients (e.g., NCT04810091) will study the potential benefit of one of these therapies, telotristat ethyl (27).

Approximately half of our surgical patients (54%) were initiated on warfarin for bioprosthetic thrombosis protection yet only completed 3 months of warfarin treatment following TV/PV replacement due to increased bleeding risk. Our experience reflects the concerns and limited use of mechanical valves that require long-term use of anticoagulation, challenging therapy in patients with abnormal coagulation profiles from liver metastasis and persistent hepatic congestion secondary to right-sided heart disease. In our population, patients with high disease liver burden and increased liver enzymes were not initially started on anticoagulation (also in the absence of clear anticoagulation guidelines for bioprosthetic right sided valves). After individualized risk/benefit discussion with the cardio-oncology team, patients with post-surgical increase in PV velocities were initiated on anticoagulation and, if velocities continued to increase, cancer treatment escalated to include advanced therapies (e.g., telotristat or PRRT). Notably, patients with CnHD may also require biopsies and tumor reduction procedures that pose additional risk while they are on anticoagulation therapy (28). Transvalvular gradients were not affected by the presence or absence of anticoagulation, favoring a NET-related rather than a thrombotic etiology for valve degeneration.

**FIGURE 6:**
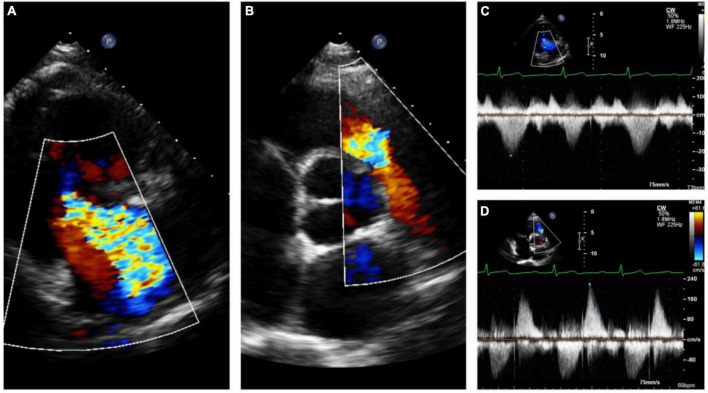
Echocardiography monitoring in carcinoid heart disease (CnHD): Pre-surgical baseline echo. Severe tricuspid regurgitation **(A)** with pulmonary valve stenosis **(B)** with corresponding continuous wave Doppler **(C, D)**.

**FIGURE 7:**
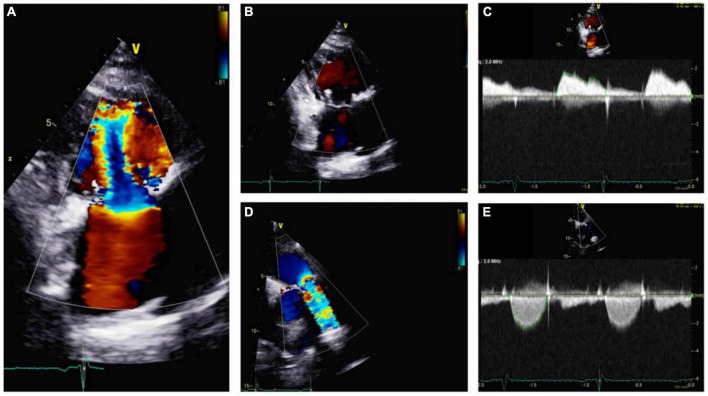
Echocardiography monitoring in carcinoid heart disease (CnHD): First follow up echo. Tricuspid valve in parasternal long axis showing mild tricuspid stenosis **(A)** and a lack of tricuspid regurgitation **(B)**; continuous wave color Doppler shown **(C)**. Pulmonic valve is shown in color Doppler **(D)** and continuous wave color Doppler, where there is evidence of increased pulmonary valve velocity **(E)**.

**FIGURE 8:**
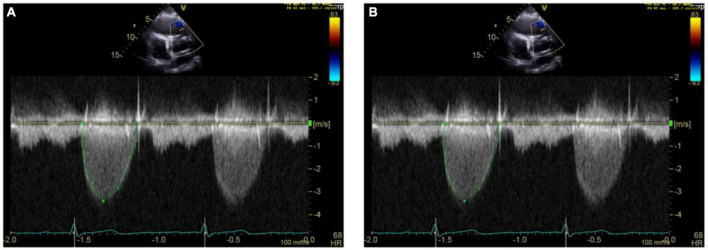
Echocardiography monitoring in carcinoid heart disease (CnHD). Second and third follow up echos. Follow up echo at 6 **(A)** and 9 **(B)** months after surgery showing higher pulmonary valve velocities.

Systematic echocardiographic monitoring played a major role in the post-surgical period. Echocardiography helped us identify the rather-unpredictable timing of the bioprosthetic valve degeneration (Figures 6–8). Pulmonary valve stenosis was the primary indicator of overall degeneration (determined by slight increases in PV velocities, V_max_ values, and the severity of valvular regurgitation) and occurred from 6 to 9 months to 2 years after surgery. One patient had a stepwise deterioration that required escalating her treatment regimen from telotristat ethyl treatment to PRRT treatment to achieve valve stabilization.

With the CVHD echocardiography scoring system parameters we have documented expected post-surgical changes in velocities, gradients, and severity levels of valvular involvement after bioprosthetic TV and PV replacement (14). Bioprosthetic TV and PV hemodynamic parameters differed significantly from pre-surgical baseline to the first post-surgical follow-up and the new baseline 3 months post-operatively, with TV and PV velocities trending differently (Table 3). Bioprosthetic TV velocities and gradients showed negative differences at the post-operative follow up compared to pre-operative, suggesting the absence of higher forward flow in diastole due to tricuspid regurgitation and valvular stenoses consistent with normal function of the bioprosthetic valve. Bioprosthetic PV gradients and velocities, however, had higher values than baseline and in almost 1/4th of patients followed an upward trend in peak/mean gradient as well as V_max_ at the second (6 months post-op) follow up as well. These climbing values, in conjunction with the visualization of bioprosthetic PV degeneration on echo imaging, suggest that bioprosthetic PVs appear to be more vulnerable than TVs to degeneration from systolic neurohormonal/serotonin wash and increased velocities. This degenerative phenomenon has been noted in other studies of explanted PV autografts and has been thought to derive mostly from myofibroblast and endothelial stress (29, 30). We acknowledge that, traditionally, carcinoid syndrome patients’ with carcinoid heart disease present, as expected, with both PV and TV deterioration, as they are off treatment or inadequately treated (e.g., with somatostatin analogs) (31). Early increase in velocities after surgery at the pulmonic valve level at almost pre-procedure levels, however, suggest the possibility of plaque deposition specifically–and in higher concentration–at the pulmonic valve level. Once carcinoid disease/carcinoid syndrome and CHD diagnosis are made and treatment of symptoms started (all patients are already on long acting somatostatin analogs), it appears that the occult amount of uncontrolled (residual) disease and now lower levels of serotonin preferentially deteriorate the PV; there is likely a role of additional right ventricular systolic flow turbulence “washing” the leaflets of the PV (Table 4).

**TABLE 4:** Follow-up echocardiographic data.

Covariate		First follow-up (New baseline)	Second follow-up	Difference	
	*n*	Mean ± SD	Mean ± SD	Mean ± SD	*P*-value^1^
LV EF^2^	18	60 (57–65)	60.5 (57–65)	0 (−4–5)	0.5331^3^
CVHD	15	77.62 ± 10.75	35.71 ± 8.95	−41.9 ± 17.88	**<0**.**0001**
TVpeak velocity	16	2.27 ± 0.54	1.53 ± 0.47	−0.74 ± 0.67	**0**.**0005**
TV peak gradient	16	22.29 ± 9.19	10.17 ± 6.78	−12.11 ± 9.9	**0**.**0002**
TV mean gradient^2^	16	7.6 (4.5–11.5)	4.2 (3.1–6.7)	−2.1 (−5.9–0.4)	**0**.**0386**^3^
PV peak velocity	15	1.76 ± 0.45	2.39 ± 0.79	0.63 ± 0.74	**0**.**0053**
PV peak gradient	15	13.19 ± 6.13	25.35 ± 14.82	12.17 ± 14.64	**0**.**0062**
PV mean gradient	15	6.77 ± 3.22	15.11 ± 8.34	8.35 ± 8.12	**0**.**0014**
Systolic blood pressure^2^	17	120 (111–134)	118 (110–125)	−15 (−27–5)	0.0694^3^
Diastolic blood pressure^2^	17	76 (72–83)	71 (63–76)	−9 (−15– −1)	**0**.**0168**^3^
Heart rate	19	77.26 ± 13.35	82.89 ± 19.67	5.63 ± 16.17	0.1464

^1^Paired *t*-test is used unless specified.

^2^Median (IQR) are presented instead of Mean ± SD.

^3^Wilcoxon signed rank test is used.

CVHD, carcinoid valvular heart disease; EF, ejection fraction; LV, left ventricular; PV, pulmonary valve; SD, standard deviation; TV, tricuspid valve.

At the first follow-up (3-months post-op; deemed the new baseline), the mean CVHD score, TV peak velocity, TV peak gradient were lower than the baseline values indicating improved heart function; changes in systolic blood pressure were not statistically significant. At the second follow-up (6 months post-op), the mean CVHD score and diastolic blood pressure values were also lower than the baseline values; however, the mean PV peak velocity, PV peak gradient, and PV mean gradient values continued to rise.

Bold values represent the statistically significant; *p*-value < 0.05.

The standard monitoring systems or algorithms for the surveillance of valve integrity, and, possibly, neurohormonal suppression with advanced therapies may improve the longevity of bioprosthetic valves in patients with CnHD. The changes in post-surgical CVHD scores offer insights into ventricular remodeling, while changes in velocities underline the importance of monitoring bioprosthetic pulmonic valves for signs of degeneration in CnHD.

### Study limitations

There are no validated scores for bioprosthetic valves in CHD patients; we contemplated the existing available scoring systems for native valves and, to have a better quantitative/qualitative tool, we decided to use the Denney score (14, 32). Because patients were asymptomatic on octreotide and lanreotide and had stable tumor burdens, uniform levels of plasma or urine 5- hydroxyindoleacetic acid were not obtained. Future studies with serial monitoring of post-surgical levels of the acid could help define the “valvular toxic” level of 5-HIAA (9). NT-proBNP could provide an early signaling role and could help post-surgical monitoring. In addition, not all patients had cardiac MRI before or after surgery, although its systematic use could have improved valvular assessment and provided separate analysis of right ventricular ejection fractions (22, 33). We had 10 patients with cardiac MRI data, with slight improvement in visualization of the pulmonic valve; these studies, however, provided no change in the severity of the stenosis/regurgitation after correlation to transthoracic echocardiography. We recognize that we could not determine the impact of possible valve deterioration peri-operatively when there existed concomitant ventricular remodeling with subsequent, expected increase in valvular velocities. Lastly, due to the small size of our study we have not captured any tricuspid valve stenosis, important pathology that requires also monitoring.

## Conclusion

Bioprosthetic degeneration is frequently encountered in patients with CnHD after valve replacement, especially in bioprosthetic pulmonary valves. More aggressive tumor and neuroendocrine hormonal control alongside systematic monitoring with standardized echocardiographic studies every 3 months after valve replacement surgery could be major keys in prevention and early identification of patients at risk for severe valve degeneration. Extended valve monitoring leading to treatment with advanced therapies may help prevent late valve degeneration driven by disease progression. PV velocities represent an early indicator of valve degeneration and should be systematically obtained. Novel neurohormonal treatments with telotristat ethyl and PRRT could show promising bioprosthetic valve stabilization role.

## Data availability statement

The raw data supporting the conclusions of this article will be made available by the authors, without undue reservation.

## Ethics statement

The studies involving human participants were reviewed and approved by MD Anderson Ethics Committee. Written informed consent for participation was not required for this study in accordance with the national legislation and the institutional requirements.

## Author contributions

All authors listed have made a substantial, direct, and intellectual contribution to the work, and approved it for publication.
