# Supplementary material for: Bioprosthetic valve monitoring in patients with carcinoid heart disease
Source: Front Cardiovasc Med. 2023 Jan 12;9:1072890. doi: 10.3389/fcvm.2022.1072890 (PMC9878394; doi:10.3389/fcvm.2022.1072890)

Supplementary Figure 1. The effect of telotristat ethyl treatment with or without peptide receptor radionuclide therapy on postsurgical pulmonary valve velocities.

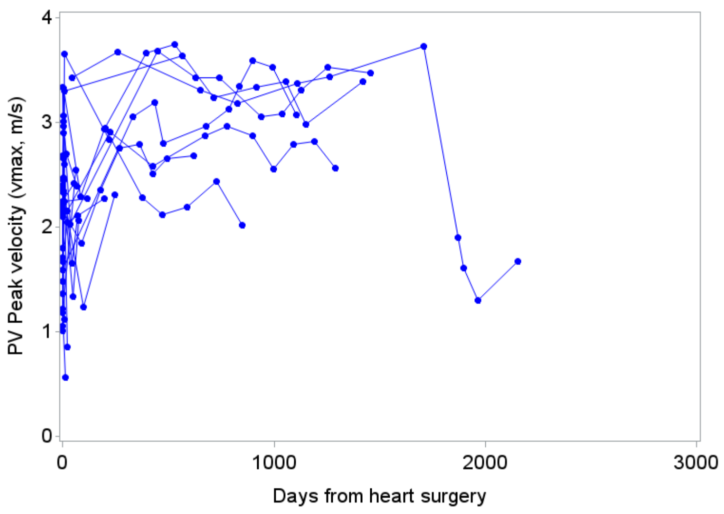

Supplement: Supplementary file 1 [file Image_1.pdf]
